# Traumatic incident reduction: A suitable technique for South African social work practice settings

**DOI:** 10.4102/hsag.v24i0.1106

**Published:** 2019-05-29

**Authors:** H. Marili Williams, Elizabeth C. Erlank

**Affiliations:** 1 Department of Social Work, University of South Africa, Pretoria, South Africa

**Keywords:** Mental health, post-traumatic stress disorder, traumatic incident reduction technique, social work, social workers, healthcare practitioners

## Abstract

It is part of South African social workers’ responsibilities to attend promptly and appropriately to victims of trauma. Overstrained and limited resources in communities influence the availability of debriefing services to traumatised community members. The purpose of this article was to elaborate on the traumatic incident reduction (TIR) technique as a suitable, short-term intervention technique for social work practice settings to address the impact of trauma effectively and timely.

A discussion on TIR is presented by contextualising and defining mental health and post-traumatic stress disorder (PTSD) and elaborating on other practice approaches, therapies, techniques and models for addressing trauma and PTSD in social work practice.

Given the excessive exposure to trauma experienced by the South African population, it is clear that further trauma counselling services are required and more effective ways must be found to empower communities to deal with trauma. Most approaches for the treatment of trauma in South Africa are specialised and resources are limited; therefore, many communities are excluded from these specialised services. The TIR technique fits appropriately within the ambit of the developmental approach, as embraced by the South African Department of Social Development.

It will be beneficial if social workers, auxiliary social workers, community leaders, community volunteers, health care workers and lay counsellors are trained in the TIR technique. Concerted efforts are necessary to empower communities in supporting themselves and developing the necessary skills to address trauma. This initiative will be consistent with the developmental approach sanctioned by the Department of Social Development.

## Introduction

Social workers in practice assist victims of trauma, and the onus is on them to improve their knowledge and skills with regard to appropriately and effectively assisting trauma victims (Suffla, Van Niekerk & Duncan 2004). Limited resources are available to social workers when they have to refer clients to various mental health facilities with the purpose of resolving clients’ mental distress resulting from trauma (Blokland 2014:75; Plüddemann et al. 2014:34). One of the main aims of social work practice is to continuously explore various evidence-based approaches to ensure effective service delivery. Traumatic incident reduction (TIR), an applied meta-psychology technique (Moore 2005:14), is introduced in this article to challenge social workers to adopt an integrated and effective trauma-related service that can be accessed by all South Africans and communities.

The authors will elaborate on post-traumatic stress disorder (PTSD) as a mental health concern in South Africa, followed by a discussion on the practice models available that deal with trauma in social work practice. A theoretical framework of TIR will be presented as well. Suggestions as to how TIR can be applied in social work practice within a multicultural environment will also be provided. This article therefore aims to illustrate a discussion of TIR and how the TIR technique can be successfully implemented in social work practice by social workers.

## Background

The South African population is exposed to various forms of crime and violence, and South Africa is presently considered as one of the most violent countries internationally (Williams et al. 2007:845). According to the United Nations Office on Drugs and Crime’s *Global Study on Homicide 2013* (2014):

… the global average homicide rate stands at 6.2 per 100 000 population, but Southern Africa (inclusive of South Africa) and Central America have rates over four times higher than that (above 24 victims per 100 000 population), making them the sub-regions with the highest homicide rates on record. (p. 22)

*South African Crime Statistics 2013–2014,* as reported by the Institute of Security Studies (ISS 2014), confirms that South Africans experience high numbers of assaults, rapes and murders with firearms.

During 2008, it was already determined that one-third of all South Africans had been exposed to some form of violence or crime-related incident (Kaminer et al. 2008:1589). Needless to say, it is impossible for any person – and/or any community – who has been exposed to traumatic events not to be affected in some way (Williams et al. 2008:211), as ‘violence damages individual mental and physical well-being, family welfare, community cohesion, and the country’s overall social, political, and economic development’ (Seedat et al. 2014:137). One of the most likely disorders coming to the fore in response to traumatic events experienced by people is PTSD, and it was noted by Edwards (2005:125) that PTSD is a significant public health problem in South Africa.

The *Diagnostic and Statistical Manual of Mental Disorders 5* (DSM-5), according to Dziegielewski (2014:5), is ‘a system that accurately identifies and classifies biopsychosocial symptoms’, and it is a classification structure that forms an important basis for assessing mental health issues. ‘Post-traumatic stress disorder’ refers to a diagnosis based on the criteria set out in the DSM-5 (American Psychiatric Association 2013), which are associated with various symptoms resulting from the traumatic event.

The symptoms of PTSD include different forms of re-experiencing the trauma (e.g. through nightmares, sensory flashbacks or involuntary images), behavioural attempts to avoid traumatic reminders, amnesia for some aspects of the trauma, emotional numbing and symptoms of hyperarousal (such as an exaggerated startled response and constant hypervigilance for danger), all of which must be present for at least 1 month and cause significant distress or impairment in functioning (American Psychiatric Association 2000 in Kaminer et al. 2008:1589; Krystal & Neumeister 2009:13).

The majority of victims experiencing trauma do not automatically develop long-lasting mental problems, and they manage to recover fully from the traumatic event; however, it does happen that some individuals experience ongoing and severe symptoms that impair their well-being and social functioning. A diagnosis of PTSD will then be appropriate (Kaminer & Eagle 2010:28).

## Trauma and post-traumatic stress disorder: A mental health concern in Africa and South Africa

The World Health Organization (WHO) Fact Sheet Number 220 (2014) defines ‘mental health’ as follows:

… a state of well-being in which the individual realizes his or her own abilities, can cope with the normal stresses of life, can work productively and fruitfully, and is able to make a contribution to his or her community. (para 2)

It is recognised by Edwards (2005:125) that ‘across Africa, catastrophic civil wars and natural disasters have been a source of trauma on a massive scale and accidents and assaults are an everyday occurrence’. Africa is known for its wars, disasters and crimes, which fuel the experiences of trauma among thousands of victims. It is highlighted by Amanyuzu-Nyamongo (2013:590) that ‘the social environment in many African countries does not nurture good mental health, mainly due to the myriad conflicts and post-conflict situations’. The author (Amanyuzu-Nyamongo 2013:590) further postulates that the status of mental health and psychosocial well-being of people is fundamentally influenced by the prevalence of and exposure to war and major disasters; in Africa an estimated 50% of refugees are suffering from a mental illness, including PTSD (Amanyuzu-Nyamongo 2013:59; Consultancy Africa Intelligence’s Public Health Unit 2013; Plüddemann et al. 2014:34).

In South Africa, the prevalence of PTSD continues to be a concern (Edwards 2005:127; Williams et al. 2007:846). The lifetime prevalence and incidence of PTSD in the general population is 1%–9% (Zungu 2013:22). The experience of trauma is not limited to the person who experiences it, but a quarter of the people who witness a traumatic event will also be at risk of developing PTSD (Van Zyl, Oosthuizen & Seedat 2008:119). Reports indicate that since the early 1990s rates of violent crime and sexual and domestic violence have increased, which makes victims vulnerable to developing PTSD, affecting the mental health status of South Africans (Kaminer et al. 2008:1589). Several research studies’ findings consistently point to the fact that the experience of violence by victims will most likely lead to a diagnosis associated with PTSD (Edwards 2005:127; Kaminer et al. 2008:1589; Sullivan et al. 2005:2); therefore, the overall mental health status of South Africans should be addressed.

It is stated by Sullivan and Stein (2012) that:

… the South African Stress and Health study has estimated that seventy five percent (75%) of adult South Africans have experienced a traumatic event, such as a physical or sexual assault, motor vehicle accident or disaster at some point in their lives. (p. 308)

Not everyone who experiences trauma will develop PTSD, but the prevalence of PTSD in South African communities is estimated at 2.3%, with a higher incidence among primary health care populations (Sullivan & Stein 2012:308). A study conducted by Peltzer (1999), with children from a rural environment, revealed that 67% of the sample had experienced a traumatic event and 8% of those developed PTSD. Studies conducted in the Western Cape also provided evidence of a high prevalence of traumatisation and PTSD among youth (Sullivan et al. 2005:2). The lack of sufficient resources, as well as the failure to ensure early detection and to provide effective treatment interventions, creates conditions for long-term chronic mental health impairment conditions (Amanyuzu-Nyamongo 2013:59).

Recently the term ‘*continuous* traumatic stress’, as opposed to ‘*post*-traumatic stress’, has surfaced in the field. This is because of the ongoing above-average rates of trauma-related incidences such as violent crime, sexual violence and trauma-related deaths experienced by South Africans (Kaminer & Eagle 2013 in Eagle 2014:3), and therefore it is concluded that trauma and PTSD will always be present in our communities. Hence, it is essential that the scope of social work practice prioritise mental health, trauma and PTSD. Social workers will have to empower themselves with optimal and effective usage of techniques when dealing with trauma victims, as well as PTSD cases, as the South African society is likely to be exposed to ‘continuous traumatic stress’ for some time to come.

It can be concluded that the status of South Africans’ mental health will remain a concern for many social workers in practice. To deal with these mental health concerns in South Africa, social workers would have to be conversant with the various practice approaches, therapies and models that are available with regard to the treatment of trauma and PTSD. The following section will discuss these options, in order to familiarise social workers with the place that TIR can fulfil.

## Practice approaches, techniques and models for addressing trauma and post-traumatic stress disorder

The treatment of trauma was already a focus point in psychotherapy during the 1800s (referring to the early work of Freud). Over the past 20 years, various therapeutic approaches have been developed and are available to social workers to mitigate and deal with the symptoms of traumatic stress disorder (Garrick & Williams 2006:1; Kaminer & Eagle 2010:80). It is also not uncommon for psychiatric medication to be prescribed for patients who display symptoms of trauma (Kaminer & Eagle 2010:81). According to Gerbode (2005:1) (in Volkman 2005:1), the most common approaches to assist in reducing symptoms of post-traumatic stress are coping and cathartic techniques.

One of these approaches is known as ‘critical incident stress debriefing’ (CISD), which was first described by Jeff Mitchell (1993) and which refers to a formal group procedure for emergency workers to address possible responses to an incident that may overwhelm the individual’s coping mechanisms (Volkman 2007:vii). The crisis intervention is brief, to prevent it worsening and to promote recovery as soon as possible (Volkman 2007:vii). The CISD approach stimulated the development of the intervention known as ‘psychological debriefing’ (PD) (Bisson et al. 2009:83 in Foa et al. 2009:83). The PD model was modified in order for it to be used in group interventions (Bisson et al. 2009:83 in Foa et al. 2009:90). The effectiveness of the CISD approach and the PD model, as well as other interventions such as psychological first aid and trauma risk management, in the prevention of PTSD and the reduction of stress after a critical or traumatic incident must still be determined through rigorous research (Bisson et al. 2009:83 in Foa et al. 2009:100).

Another approach offered in the treatment of PTSD and trauma is cognitive behaviour therapy (CBT). According to Hofmann et al. (2012), CBT addresses maladaptive cognitions that cause emotional and behaviour problems. These maladaptive cognitions may include general beliefs about the self, others and the world. The use of CBT has presented the most promising outcomes with regard to the treatment of distress and in the prevention of long-term psychopathology (Bisson et al. 2009:83 in Foa et al. 2009:100).

The approaches that are popular in South Africa are CBT (and under its auspice, prolonged exposure), cognitive processing therapy, stress inoculation therapy and cognitive therapy (CT) (Kaminer & Eagle 2010:89). Narrative, psychodynamic and psychoanalytic therapies have also been employed in South Africa to assist clients (Kaminer & Eagle 2010:94). The Wits trauma model, developed by the University of the Witwatersrand, is a popular tool to treat traumatised persons and yields positive outcomes (Kaminer & Eagle 2010:95). This model is integrative, uses cognitive behavioural techniques and has psychodynamic underpinnings (Kaminer & Eagle 2010:95).

Other approaches and techniques that need to be mentioned are eye movement desensitisation and reprocessing, TIR, visual kinaesthetic dissociation and thought field therapy, but evidence could not be found that these interventions are widely used in South Africa (Kaminer & Eagle 2010:95). According to Kaminer and Eagle (2010:96), empirical research is still needed on these therapies, but it seems that they are very promising in the alleviation of trauma symptoms (Kaminer & Eagle 2010:96). There is consensus among trauma service providers that a strong therapeutic relationship with the client is vital (Kaminer & Eagle 2010:108). The most recommended techniques for trauma counselling are anxiety management, CT, exposure therapy and psycho-education (Kaminer & Eagle 2010:109).

It is clear that a number of practice approaches, techniques and models for addressing trauma are available to social workers; however, because of the high number of caseloads (Kasiram 2009:647), it is critical that social workers find the most effective intervention strategy to assist clients exposed to trauma. It is argued that TIR is a practical and effective technique to implement within social work practice settings and that it yields positive results (Valentine 2000:1–15). A discussion of TIR is introduced in the following section, with special reference to social work practice.

## Introducing traumatic incident reduction in social work practice

The TIR approach, as a suitable technique for social work practice settings, is introduced here, with the intention of expanding the menu of trauma intervention techniques available to social workers.

Traumatic incident reduction is registered at the Substance Abuse and Mental Health Services Administration (SAMHSA) and the National Register of Evidence-based Programs and Practices (NREPP). An agency within the US Department of Health and Human Services, SAMHSA leads public health efforts to advance the behavioural health of the nation. The mission of SAMHSA is to reduce the impact of substance abuse and mental illness on US communities. The NREPP is an online, searchable database of interventions designed to promote mental health or to prevent or treat substance abuse and mental disorders. The goal of the NREPP is to encourage wider adoption of evidence-based interventions and to help those interested in implementing an evidence-based intervention to select one that best meets their needs. The interventions listed by the NREPP need to meet certain criteria to be placed on the registry; TIR is one of the interventions that can be found on the registry.

According to Volkman (e-mail to author 2015), the president of Applied Metapsychology International and the Traumatic Incident Reduction Association (TIRA), 750 South African practitioners (inclusive of social workers) are registered on TIRA’s database. It is, therefore, significant progress that South African practitioners are empowering themselves with techniques to mitigate the effects of trauma, thus assisting victims of trauma.

Traumatic incident reduction, an applied meta-psychology protocol, was developed by Frank Gerbode in the 1980s, to resolve trauma in trauma victims (Gerbode 1995:387). Traumatic incident reduction is explained by Moore (2005:14) (in Volkman 2005:14) as ‘a regressive desensitization procedure for reducing or eliminating the negative residual impact of traumatic experience’, and therefore TIR is recognised as a directive and client-centred technique (Moore 2005:14 in Volkman 2005:14). Gerbode (1995:588) states that meta-psychology, the framework under which TIR was developed, is the study of the person and his or her abilities; the origin, structure and function of the mind; and the relationship between the person, mind and physical universe. The rules of facilitation are guided by a strict code that forms an integral part of the TIR protocol. The rules of facilitation are, to a large extent, similar to the propositions presented by Carl Rogers (Gerbode 2013:254; Rogers 1980:113); Gerbode (1995:387) mentions that ‘although some or all of them (the rules of facilitation), may seem obvious or simplistic, their importance cannot be overstressed’. It is apparent that the majority of unsuccessful facilitating interventions can be traced back to a violation of these rules (Gerbode 2013:253).

The therapeutic principles followed in social work, the person-centred approach and the propositions by Carl Rogers feature very prominently in both meta-psychology and the TIR approach (Gerbode 1995:13; Grobler & Schenk 2009:12–106). The applied meta-psychology framework, with its rules of facilitation, and the person-centred approach in social work provide a fitting framework within which TIR can be effectively applied. Traumatic incident reduction combines the person-centred approach – where the client is the authority of his or her experience – with a highly structured approach that clearly defines the roles of the client and the facilitator as well as the protocols that should be followed (Gerbode 2006:154).

Traumatic incident reduction is described as a rapid method (as compared to traditional therapy) of effectively reducing traumatic stress caused by emotionally and/or physically painful events in the past. It involves the client re-experiencing past traumas in a completely safe environment (Volkman 2005:304). The recovery process, according to Herman (1997:133–196), of trauma may be conceptualised in three stages: establishing safety and stabilisation; retelling the story of the traumatic event and reconnecting with others. The treatment of post-traumatic disorders must be appropriate to the client’s stage of recovery (Herman 1997:145). One of the rules of facilitation in the TIR protocol is to create a suitable and safe space for the client, which confirms the first phase of Herman’s stages of recovery (Gerbode 2013:253). The second phase of Herman’s stages of recovery is retelling the story of the traumatic event, which overlaps with the TIR protocol as a regressive and imaginal desensitisation procedure in the treatment of trauma (Moore in Volkman 2005:15). The reconnection phase deals with creating future goals, reclaiming his or her life and developing new relationships (Herman 1997:196). The viewpoint of meta-psychology supports this phase of improving the client’s well-being and functioning (Day 2007:2 in Volkman 2007:49).

The protocols followed in TIR have universal and multicultural application, which is beneficial and valuable in the multicultural setting found in South Africa (Gerbode 2006:154, 2013:283). A part of the TIR protocol requires the retelling of the traumatic event, which is compatible with the traditional storytelling practices of the African and Asian cultures (Gerbode 2013:275, Kaminer & Eagle 2010:91). The rules of facilitation, also a part of the TIR protocol, are culturally friendly, because the interpretation and meaning of the traumatic event are exclusively left to the client, with no interpretation from the facilitator’s side (Gerbode 2013:254). These aspects of the TIR protocol allow and accommodate any cultural meaning that the client may attach to the traumatic incident.

**FIGURE 1:**
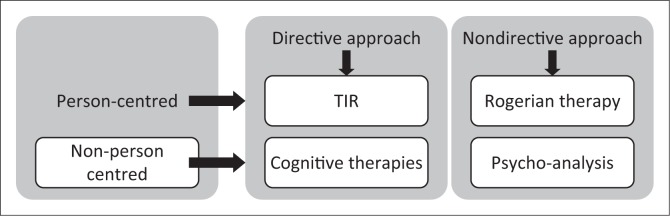
Illustration of traumatic incident reduction in relation to other therapies.

Figure 1 illustrates the differences in the approaches in relation to the TIR approach. Traumatic incident reduction is a person-centred, directive approach. Rogerian therapy, on the other hand, values a person-centred, non-directive approach. The CTs are non-person-centred, more directive approaches, while psycho-analysis has a non-person-centred, non-directive approach.

Research evidence regarding the effectiveness of TIR as a trauma-resolution technique was conducted by Valentine (2000:1–15) among incarcerated females at the Tallahassee Federal Correctional Institute in Florida. An experimental outcome study examined the effectiveness of TIR in treating trauma-related symptoms of 123 female inmates who were victims of interpersonal violence. The study specifically examined the effectiveness of TIR on symptoms of PTSD, depression, anxiety and low expectancy of success. Pretests, post-tests and a 3-month follow-up were done using a PTSD symptoms scale, the Beck Depression Inventory, Clinical Anxiety Scale and the Generalized Expectancy for Success Scale. A statistically significant decrease was documented in PTSD, depression and anxiety symptoms (Valentine 2000:1–15).

The effectiveness of applying TIR with children who had experienced trauma was determined, and it involved having the child discuss the traumatic experience several times (Descilo 2005:167–185; Furze 2005:201–206; Volkman 2007:91–153). Appropriate play therapy techniques can also be employed with TIR (Hamblen 2001 in Volkman 2007:169). The reader is reminded that TIR is a regressive and imaginal desensitisation procedure technique in the treatment of trauma (Moore 2005:14 in Volkman 2005:15). The biggest factor in determining whether a child (or an adult) is able to make use of TIR is his or her ability to focus attention on the material being addressed (Volkman 2005:165).

## Traumatic incident reduction as a technique in social work practice settings

Practising social workers are required to have a comprehensive understanding and knowledge of and experience in the application of appropriate theoretical frameworks, theories, models and approaches pertinent to the methods applied in social work (case work, group work, community work).

The social worker trained in applying the TIR techniques will follow similar processes to those practised in the field of social work, like assessing in a person-centred way (to determine the needs of the client), determining the existence of prior traumatic events and, most importantly, whether the client is interested in addressing the traumatic events (Gerbode 2013:261–263). Often, there are other needs of the client that must be attended to first, before any trauma counselling may be possible (Gerbode 2013:330).

Applying the TIR technique requires the assurance of a safe space, where the client can feel secure to share the traumatic experiences with the social worker (Gerbode 2013:253). In South Africa, the tendency of prolonged traumatisation (continuous traumatic stress) is part of many economically disadvantaged communities, which involves, for example, daily experiences of violence in the community (Kaminer & Eagle 2010:48). Therefore, to create a safe space for trauma intervention may be difficult; there may not necessarily be a ‘post-traumatic’ time – when the client has the opportunity to adapt to the traumatic effects – before the next traumatic experience may occur (Kaminer & Eagle 2010:49).

The duration of a typical TIR session is one to four hours, and an average session is usually not longer than one and a half hours, while more intense cases may take up to two and a half hours (Gerbode 2006:163).

The process of applying TIR entails the following (Gerbode 2006:159–161): the client is asked questions to ascertain when the incident happened (when), where the incident happened (where) and how long the incident lasted (length); the client will then be asked to go to the starting point of the incident (begin) – at this time, the client has to explain what he or she was aware of at the exact starting point of the incident (aware), is asked to share his or her version of events up to the end of the incident (go) and to articulate what happened (tell). The social worker (facilitator) will listen without commenting on all that the client shares until the client is done. Usually the client will narrate the details of the incident, share his or her reactions and feelings and any other thoughts about the incident or anything that comes to mind (Gerbode 2006:161).

A TIR session is characterised by the client starting at a moderate level of distress and, as the session continues, the level of distress increases until it reaches a peak. The intensity then decreases, until a plateau is reached and the client reaches the end point. The three important signs of reaching the end point are notable in the client: positive indicators, extroversion and realisations (Gerbode 2006:161).

Because of the limited availability of resources for social work services in communities, social workers will find that the TIR technique is an effective way of addressing trauma in practice, as it yields positive results in a relatively short period of time. Providing training to social workers in this approach will increase their effectiveness in dealing with trauma and may avert the need for further client referrals to other mental health services. It should be noted that the TIR technique can be implemented not only by trained social workers but also auxiliary social workers, community leaders, community volunteers and lay counsellors, who can be trained in this technique, thus increasing the availability of trauma debriefing services to community members – particularly where communities have limited access to trauma counselling.

## Conclusion

The availability of resources is an integral problem, not just for underdeveloped communities but for the social workers in social work practice as well (Alpaslan & Schenck 2012:367). Given the excessive exposure to trauma experienced by the South African population, it is clear that more trauma counselling services are required and more effective ways must be found to empower communities to deal with trauma. Most approaches for the treatment of trauma in South Africa are specialised and resources are limited; therefore, many communities are excluded from these specialised services.

The TIR technique fits appropriately within the ambit of the developmental approach, as embraced by the South African Department of Social Development. The developmental approach entails holistically planned interventions, with the focus on person-centredness and on addressing social concerns in a strength-based and systems approach manner to warrant social change (Perumal 2015:64).

The implementation of the TIR technique by social workers will ensure that communities in dire need of appropriate resources can at least receive trauma debriefing as and when the need arises. The TIR technique is a modest person-centred, directive and structured trauma approach that has proven positive outcomes (Gerbode 2006:165) and can be utilised within different community settings.

## Recommendations

The following recommendations are suggested:

- Train social workers in TIR, thus empowering them to support victims of trauma effectively and without delay.
- Provide training to auxiliary social workers, community leaders, community volunteers and lay counsellors in the TIR technique (by the trained social workers), which will escalate the availability of trauma debriefing services to already traumatised communities in a shorter space of time (Durkin 2012:25).
- Empower communities in supporting themselves and developing the necessary skills to address trauma, which is consistent with the developmental approach supported by the Department of Social Development (Perumal 2015:63).

## References

[CIT0001] Alpaslan N. & Schenk R, 2012, ‘Challenges related to working conditions experienced by social workers practising in rural areas’, *Social Work/Maatskaplike Werk* 48(4), 367–386.

[CIT0002] Amanyuzu-Nyamongo M, 2013, ‘The social and cultural aspects of mental health in African societies’, *Commonwealth Health Partnerships* 2013, 59–63.

[CIT0003] American Psychiatric Association, 2013, *Diagnostic and statistical manual of mental disorders (DSM-5®)*, American Psychiatric Association Publication, Arlington, VA.

[CIT0004] Bission J.I., McFarlane A.C., Rose S., Ruzek J.I. & Watson P.J, 2009, ‘Psychological debriefing for adults’, in Foa E.B., Keane T., Friedman M. & Cohen J.A. (eds.), *Treatment guidelines for post-traumatic stress disorder*, Guilford Press, New York.

[CIT0005] Blokland L.M.E, 2014, ‘Mental health care in Mamelodi: Disadvantaged geographical positioning in a South African township’, *De Jure Journal* 47(2), 175–188.

[CIT0006] Consultancy Africa Intelligence’s Public Health Unit, 2013, *The silent crisis: Mental health in Africa*, Creamer Media, Johannesburg.

[CIT0007] Day N.L, 2007, ‘TIR as a companion to CISM/CISD’, in Volkman M.K. (ed.), *Children and traumatic incident reduction: Creative and cognitive approaches*, vol. 2, Loving Healing Press, Ann Arbor, MI.

[CIT0008] Descilo T, 2005, ‘Children and TIR’, in Volkman V.R. (ed.), *Beyond trauma: Conversations on traumatic incident reduction*, 2nd edn, AMI Press, Ann Arbor, MI.

[CIT0009] Dziegielewski S.F, 2014, *DSM-5 in action*, 3rd edn, John Wiley & Sons, Inc., Hoboken, NJ.

[CIT0010] Durkin J, 2012, ‘Empowerment versus treatment for trauma victims’, *Occupational Health* 64(11), 24–25.

[CIT0011] Eagle G, 2014, ‘From evolution to discourse: Key conceptual debates in the history and study of traumatic stress’, *Psychology in Society* 47, 1–20.

[CIT0012] Edwards D, 2005, ‘Post-traumatic stress disorder as a public health concern in South Africa’, *Journal of Psychology in Africa* 15(2), 125–134.

[CIT0013] Foa E.B., Keane T., Friedman M. & Cohen J.A. (eds.), 2009, *Treatment guidelines for post-traumatic stress disorder*, Guilford Press, New York.

[CIT0014] Furze P, 2005, ‘Integrating therapies’, in Volkman V.R. (ed.), *Beyond trauma: Conversations on traumatic incident reduction*, 2nd edn, AMI Press, Ann Arbor, MI.

[CIT0015] Garrick J. & Williams M.B. (eds.), 2006, *Trauma treatment techniques – Innovative trends*, The Haworth Maltreatment & Trauma Press, Binghamton, NY.

[CIT0016] Gerbode F.A, 1995, *Beyond psychology: An introduction to metapsychology*, 3rd edn, AMI Press, Ann Arbor, MI.

[CIT0017] Gerbode F.A, 2005, ‘Critical issues in trauma resolution’, in Volkman V.R. (ed.), *Beyond trauma: Conversations on traumatic incident reduction*, 2nd edn, AMI Press, Ann Arbor, MI.

[CIT0018] Gerbode F.A, 2006, ‘Traumatic incident reduction: A person-centered, client-titrated exposure technique’, *Trauma Treatment Techniques: Innovative Trends* 12(1/2), 151–167. 10.1300/J146v12n01_08

[CIT0019] Gerbode F.A, 2013, *Beyond psychology: An introduction to metapsychology*, 4th edn, AMI Press, Ann Arbor, MI.

[CIT0020] Grobler H. & Schenk R, 2009, *Person-centered facilitation: Process, theory & practice*, 3rd edn, Oxford University Press, Cape Town.

[CIT0021] Herman J, 1997, *Trauma and recovery*, 2nd edn, Basic Books, New York.

[CIT0022] Hofmann S.G., Asnaani A., Vonk I.J.J., Sawyer A.T. & Fang A, 2012, ‘The efficacy of cognitive behavioral therapy: A review of meta-analyses’, *Cognitive Therapy Research* 36, 427–444. 10.1007/s10608-012-9476-1 23459093 PMC3584580

[CIT0023] Institute of Security Studies (ISS), 2014, South African Crime Statistics 2013/2014. Factsheet: South Africa’s official crime statistics for 2013/14, Researched by the Institute for Security Studies and Africa, viewed 15 November 2019, from https://Africacheck.org/factsheets/factsheet-south-africas-official-crime-statistics-for-201314/#sthash.U2OFyfJj.dpuf.

[CIT0024] Kaminer D. & Eagle G, 2010, *Traumatic stress in South Africa*, 1st edn, Wits University Press, Johannesburg.

[CIT0025] Kaminer D., Grimsrud A., Myer L., Stein D. & Williams D, 2008, ‘Risk for posttraumatic stress disorder associated with different form of international violence in South Africa’, *Social Science Medicine* 67(10), 1589–1595. 10.1016/j.socscimed.2008.07.023 18774211 PMC2610682

[CIT0026] Kasiram M, 2009, ‘The emigration of South African social workers: Using social work education to address gaps in provision’, *Social Work Education* 28(6), 646–654. 10.1080/02615470903027363

[CIT0027] Krystal J.H. & Neumeister A, 2009, ‘Noradrenergic and serotonergic mechanisms in the neurobiology of posttraumatic stress disorder and resilience’, *Brain Research* 1293, 13–23. 10.1016/j.brainres.2009.03.044 19332037 PMC2761677

[CIT0028] Mitchell J.T, 1983, ‘Critical incident stress debriefing (CISD)’, *Journal of Emergency Medical Services* 13(11), 49–52. https://www.ncbi.nlm.nih.gov/pubmed/10258348 10258348

[CIT0029] Moore R.H, 2005, ‘Psychological foundations of TIR’, in Volkman V.R. (ed.), *Beyond trauma: Conversations on traumatic incident reduction*, 2nd edn, AMI Press, Ann Arbor, MI.

[CIT0030] Perumal I.N, 2015, ‘We continue to “manage”: A transformational leadership perspective on social work management in the NPO sector in South Africa?’, *International Journal of Social Work and Human Services Practice* 3(2), 63–70.

[CIT0031] Peltzer K, 1999, ‘Posttraumatic stress symptoms in a population of rural children in South Africa’, *Psychological Reports* 5(2), 646–650. https://journals.sagepub.com/doi/abs/10.2466/pr0.1999.85.2.646 10.2466/pr0.1999.85.2.646 10611795

[CIT0032] Plüddemann A., Morojele N., Myers B., Townsend L., Lombard C.J., Williams P.P. et al., 2014, ‘The prevalence of risk for mental health problems among high school students in the Western Cape Province’, *South Africa, South African Journal of Psychology* 44(1), 30–35. 10.1177/0081246313516264

[CIT0033] Rogers C.R, 1980, *A way of being*, 1st edn, Houghton Mifflin Company, New York.

[CIT0034] Seedat M., Van Niekerk A., Suffla S. & Ratele K, 2014, ‘Psychological research and South Africa’s violence prevention responses’, *South African Journal of Psychology* 44(2), 136–144. 10.1177/0081246314526831

[CIT0035] Suffla S., Van Niekerk A. & Duncan N, 2004, ‘Crime, violence and injury prevention in South Africa: Developments and challenges’, Medical Research Council-University of South Africa Crime, Violence and Injury Lead Programme, pp. 145–157.

[CIT0036] Sullivan S. & Stein D.J, 2012, ‘Dealing with post-traumatic stress disorder in general practice’, *South African Family Practitioner* 54(4), 308–311. 10.1080/20786204.2012.10874240

[CIT0037] Sullivan S., Kaminer D., Seedat D. & Stein D.J, 2005, ‘Assessing post-traumatic stress disorder in South African adolescents: Using the child and adolescent trauma survey (CATS) as a screening tool’, *Annals of General Psychiatry* 4(1), 2 10.1186/1744-859X-4-2 15845137 PMC1088008

[CIT0038] United Nations Office on Drugs and Crime Global Study on Homicide, 2014, *United Nations Publication*, 14(4), 1, United Nations Publications, Austria.

[CIT0039] Valentine P.V, 2000, ‘Traumatic incident II: Re-traumatized women inmates: Maria’s story’, *Journal of Offender Rehabilitation* 31(3/4), 17–27. 10.1300/J076v31n03_02

[CIT0040] Van Zyl M., Oosthuizen P.P. & Seedat S, 2008, ‘Post-traumatic stress disorder: Undiagnosed cases in a tertiary inpatient setting’, *African Journal of Psychiatry* 11(2), 119–122. 10.4314/ajpsy.v11i2.30263 19582329

[CIT0041] Volkman V.R, 2005, *Beyond trauma: Conversations on traumatic incident reduction*, 2nd edn, AMI Press, Ann Arbor, MI.

[CIT0042] Volkman M.K, 2015, Statistics of South African practitioners of TIR, e-mail to Dr. E.C. Erlank, 07 May 2015, marian@tir.org.

[CIT0043] Volkman M.K. (ed.), 2007, *Children and traumatic incident reduction: Creative and cognitive approaches*, vol. 2, Loving Healing Press, Ann Arbor, MI.

[CIT0044] Williams S.L., Williams D.R., Stein D.J., Seedat S., Jackson P.B. & Moomal H, 2007, ‘Multiple traumatic events and psychological distress: The South Africa stress and health study’, *Journal of Traumatic Stress* 20(5), 845–855. 10.1002/jts.20252 17955545 PMC3269889

[CIT0045] Williams D.R., Herman A., Stein D.J., Heeringa S.G., Jackson P.B., Moomal H. et al., 2008, ‘Twelve-month mental disorders in South Africa: Prevalence, service use and demographic correlates in the population-based South African stress and health study’, *Psychological Medicine* 38(2), 211–220. 10.1017/S0033291707001420 17903333 PMC2718686

[CIT0046] World Health Organization. Fact Sheet N*220, 2014, *Mental health: Strengthening our response*, viewed 29 March 2016, from http://www.who.int/mediacentre/factsheets/fs220.

[CIT0047] Zungu L.I, 2013, ‘Prevalence of post-traumatic stress disorder in the South African mining industry and outcomes of liability claims submitted to Rand Mutual Assurance Company’, *Occupational Health Southern Africa* 19(2), 22–26.

